# Industrial development and urban spatial planning practices: The case of Galan and Dukem cities in Ethiopia

**DOI:** 10.1016/j.heliyon.2023.e17554

**Published:** 2023-06-26

**Authors:** Melaku Tanku, Berhanu Woldetensae

**Affiliations:** a Ethiopian Institute of Architecture, Building Construction, and City Development (EiABC), Addis Ababa University, Addis Ababa, Ethiopia; b Ethiopian Institute of Architecture, Building Construction and City Development (EiABC), Addis Ababa University, Addis Ababa, Ethiopia

**Keywords:** Industrialization, Spatial planning, Urbanization, Sustainable development, Ethiopia

## Abstract

Urbanization in developing countries like Ethiopia is taking place at an unprecedented rate. This, coupled with the rapid industrialization process, puts enormous pressure on preparing and implementing city spatial plans. As a result, most development in many cities is taking place without clear planning guidelines and lacks the institutional capacity to realize effectively the urban spatial plans. Galan and Dukem cities, located near the capital Addis Ababa, have attracted substantial industrial development investment in recent decades. This article examines the nexus between urban spatial planning and industrial development by analyzing the current urban structure plans of the two cities using institutionalist and political economy approaches. The research methodology involves key informant interviews, an expert survey, personal observation, and reviewing secondary sources. The data were analyzed using descriptive statistics and multivariate Exploratory Factor Analysis (EFA). The EFA identifies three latent variables that affect urban spatial planning performances, including the level of institutions' and actors' involvement, the state of spatial plan implementation, and the lack of public participation in planning and implementation processes. These three dimensions explain 75% of the total variations, with each accounting for 35%, 24%, and 16% respectively. The study argues that the lack of planned investment allocation and poor urban spatial plan-making and implementation hinder sustainable urban development and result in haphazard development. The successful implementation of urban spatial plans requires the involvement of various actors and strong institutional support. Therefore, urban spatial decisions need to receive policy attention to ensure efficient and sustainable urban and industrial development.

## 1 Introduction

Urbanization in developing countries like Ethiopia is taking place at an unprecedented rate. This, coupled with the rapid industrialization process, puts enormous pressure on the preparation and implementation of city spatial plans. That is why scholars like [[1], [2], [3]] argue that spatial planning and implementation require carefully designed multi-level governance arrangements. In a weak planning system, central government policies are often spatially blind, and implementing agencies find it challenging to translate policy objectives to the local circumstances [4]. For urban spatial planning to play a pivotal role in a sustainable spatial configuration, it must be supported by institutions. Developing accountable local institutions in cities addresses the problems associated with rapid urban growth in developing countries [5]. Hence, more substantial regulatory power must combine with investment to create properly planned cities [6]. Regulations must also address disparity and economic growth in cities [7].

The relationship between urban spatial planning, human life, and society is intricate and far-reaching. Urban spatial planning influences the physical urban environment, including the location of industries [[8], [9], [10]], land allocation [2,[11], [12], [13]], arrangement of buildings [14], infrastructure networks [15], and urban parks [16,17], and can significantly impact inhabitants quality of life [8,[18], [19], [20]]. Thoughtful urban spatial planning can enhance urban residents' quality of life and promote sustainability [3,13,18,[21], [22], [23], [24], [25]]. Conversely, inadequate urban spatial planning may lead to haphazard settlement [20,26,27], adversely affecting residents' livelihood and opportunities [[28], [29], [30], [31], [32]].

The primary mission of spatial planning is to maximize the use of space and spatial resources [3,6,21,33]. Unplanned urban development continued to increase in many developing countries amid soaring urban growth [34]. The critical question in Ethiopia's industry development and urban spatial planning is how far macro-level policies are integrated with territorial elements. Changes in land use associated with economic development demand policies help guide land use [11]. Land use changes affect people's lives [27]. Hence, a comprehensive intervention is required to satisfy the diverse desires of low-income people [7]. The soft focus on the urban sectors made cities face a complex range of challenges, including haphazard development, weak urban spatial plan implementations, inefficient use of urban land, and land-use changes [12,18,20,26]. The inability of institutions to address challenges in urban centers resulted in massive costs [5].

Urban spatial plan implementation problems are often associated with governance [2,13,20,22], lack of public participation [19,35,36], and the high dependence of the local government on the central government [21,33,37,38]. Decisions on plan-making and active involvement of relevant institutions and the public are important steps in urban spatial planning [2,19,21,35,39]. Many urban centers in Ethiopia do not possess the organizational and administrative capabilities and legal frameworks to undertake local economic development and efficiently deliver urban services [[40], [41], [42]]. Urbanization in Ethiopia is thought to create new conditions and opportunities for economic development and needs improvements in urban planning [37,43]. Galan and Dukem cities' spatial plans are prepared by a regional planning institution called Oromia Urban Plan Institute (OUPI) [44,45], albeit various studies [12,20,31] indicated that these plans have little link to reality. The poor performance of planning implementation is often a result of the inconsistency between land-use selection and planning control [3]. The Ethiopian government sought to strengthen its power in land-use planning [46], but changes to land-use plans are rarely publicized before implementation [11]. This has been hindered partly by rapid urbanization and limited government capacity to monitor and enforce federal and plan guidelines [46]. In countries with weak planning systems, plans are not well linked to an overall development strategy [4]. The urban plan and land allocation should be disclosed to the public through diverse channels [47].

The spatial planning system to integrate the impact of space is limited by the weakness of the conceptions of socio-spatial relations [1]. Lack of space consideration and spatial planning framework in the development process of Ethiopian cities has been common practice [48]. Most city-wide master plans in Africa have provided a very long to-do list and are thus economically impracticable [27]. Unplanned urbanization has not been an uncommon phenomenon in the study of cities [12,20,28]. In Addis Ababa and its surroundings, urban growth has been happening in an unplanned manner [30]. The population is proliferating and is expected to soar substantially in the future. This poses several opportunities and challenges for Addis Ababa and the study cities. Besides, Addis Ababa has strong interdependencies/linkages with the study cities in terms of social, environmental, infrastructure, and economic aspects [20,28,29,49]. Yet Addis Ababa and these cities have different urban-development stages [23]. This will significantly affect the needs of its surrounding localities [28,29,31].

Two related questions were addressed in the study. These are how industrial development is implemented, where urban spatial plan-making processes are highly centralized, and how urban spatial planning and implementation are carried out in such contexts. Several studies have primarily investigated urban spatial planning and implementation inconsistencies [3,13,18,21,36]. Nevertheless, industrial development and urban spatial planning practices in the context of a centrally planned structure plan are barely mentioned in the literature. This study, therefore, intends to fill this gap. The study considers institutions and actors' involvement, urban land use and spatial planning implementation, and the level of public participation used as an indicator of the performances of urban spatial planning practices [6,50]. It was based on the eclectic composited institutionalist and political economy conceptual frameworks. Applying institutional and political economy approaches gives a new insight to explain the intricate relationship between urban spatial plan-making and implementation processes and industrial development. In doing so, it aims to shed some light on the complex relationship between urban spatial plans and industrial development nexus. Following this introduction, Section 2 provides a conceptual framework for the study together with the urban spatial planning practices in Ethiopia; Section 3 briefs methods and descriptions of the study area; Section 4 is devoted to results and discussion. This paper concludes with a summary and recommendations for future research.

## 2 Literature review

### 2.1 A conceptual framework

The ‘new institutionalism’ provides an essential resource for understanding urban governance dynamics and often is associated with the disciplines of economics, political science, and sociology [[51], [52], [53]]. Hence, it is disciple-based and cannot be seen as a single discipline theoretical perspective [53,54]. Scholars [1,[51], [52], [53], [54], [55], [56], [57], [58]] have highlighted the significance of the institutionalist approach in spatial planning. The institutional approach emphasizes the variety of stakes that people have in their local communities and the variety of methods that we might make claims for policy consideration [55]. There are different definitions for the term “institution”. *Institutions* are humanly devised constraints that structure political, economic, and social interaction and consist of informal and formal rules [59].

An institution is not understood as an organization but as an established way of addressing specific social issues [1]. Ref. [59] defines organizations as; groups of individuals bound by some common purpose to achieve objectives. *Institutions* are rules defining the right to do certain things [51]. They are a kind of ‘soft infrastructure’ of the governance of social life [52]. Institutionalists acknowledge that humans are reflective beings. As a result, we decide what to accept or reject within our structured social embeddedness [1]. The institutionalist analysis focuses on interactions, not decisions per se [52]. Structural properties formulated in rules and resources are mediums and outcomes of individual actions and interactions [56]. Urbanization is a process that necessitates clear management guidance and an institutional role [60], and strengthening urban institutions now while urbanization levels are low will be essential for urbanization's success in Ethiopia [61]. Institutions are indispensable for successful urban plan implementation [36].

Both formal and informal institutions help in the realization of urban spatial plans. According to Ref. [51], the former is impersonal, albeit the latter informal is never impersonal. Ref. [59] argued that informal institutions are influential in explaining economic changes. Ref. [53] notes that government or markets offer the framework within which planning functions. Other institutions, rooted in cultural norms also offer the milieu for planning. Ref. [52] also stated that institutions are expressed in both formal rules and informal norms. In developing countries, the importance of informal institutions is indispensable. Ref. [51] explains that in developing countries, a diversity of informal institutional arrangements, including the organization of informal power in patron-client organizations, sustain the distributive requirements of all-powerful groups. Ref. [62] stated that the role of informal institutions in managing cities is immense in Sub-Saharan Africa as many formal institutional structures failed to deliver urban services.

From an institutionalist perspective, places are social constructs, given identity and infused with value through the experience of living, working, and doing business in them [1]. The spatial planning systems provide an arena where different policy concerns are integrated regarding their impact on space [6]. Arguably institutions make planning possible; without them, there would be no planning [6,63]. Countries with well-established institutions can readjust their spatial plans frequently, confirming that they benefit from planning infrastructure that most stakeholders can understand and apply. Whereas, in countries with weak planning systems, implementing planning practices is challenging [4]. In contexts with solid institutions, the self-interest of politicians and bureaucrats has less power, and actions are geared towards growth-promoting policies. In weak institutions, officials pursue their desire and rarely aim at the common good [9].

The outcome of urban spatial planning is good enough depending on the strength of the institutional framework on which it is anchored [18]. By reducing uncertainty, institutions make our expectations more reliable [63]. Creating institutional frameworks and reinforcing existing ones support sustainable industry and urban development. However, the capacity to enforce urban planning regulations needs to be improved in many developing countries [6,22,64]. The degree of achievable enforcement depends on the firmness of resistance to the enforcement of specific institutions [51]. The ‘soft infrastructure’ of governance is vital in shaping material development, attitudes, and identities, and the experience of ‘place’ is indispensable. It influences the analysis of urban spatial decisions [52]. The institutional context for urban planning significantly affects its forms and outcomes [6,65]. A study by Ref. [9] concluded that if the right institution does not support the development process, it resulted in resource misuse and rent-seeking. Ref. [66] further added that ‘knowledge’ and ‘incentive’ problems are the two major challenges with the government policy-making process. Although letting economic decisions for the market for a righteous outcome, this might not be realized due to politicians' monolithic thinking and rent-seeking or incentive problems.

The global south cities have received little attention from the political economy perspective and are primarily seen as locations for development intervention [7]. A political economy approach that considers urban political bargaining environments is indispensable for explaining urban development consequences [34,65]. Planning can be a space of political contestation where diverse political ideas are entertained [67]. However, urban planning discourses in developing countries need a focus on incorporating politics into planning methods [34]. Most often, inherited planning techniques and approaches in the global south have remained unchanged, albeit the setting in which they function has altered largely [58]. Political economy approaches are important when analyzing the effectiveness of urban planning and development regulation [34].

Urban planning is indispensable for the sustainability of cities. The political system can determine the main ideas of planning [67]. Many development organizations have considered implementing urban plans as a technical, apolitical exercise [34]. Yet, urban planning practice is deeply political [55,67], the state is the leading actor in creating legislation and is responsible for the plan implementations gap [2], and political ideology shapes how planning is managed [39]. The need for understanding planning in a context of conflicting rationalities and the role of power in shaping urban spaces is indispensable [15,57,68]. Urban activities, whether economic, social, or any other influenced and guided by politics [69,70]. Political interference with the activities of city planners has direct and indirect effects on the effectiveness of urban spatial planning [34] and this could make planners frustrated with their jobs [39]. It is challenging to separate power in the planning system as planning is a vital tool through which government manages spatially defined territories [57]. Regime theorists acclaim that public-private sector collaboration plays a vital role in city economic development and decision-making [71].

Fast industrialization and global capital movement in recent periods are influencing the role of planners and planning actions. Especially to attract foreign direct investments, central governments expanding industrial zones often urge local governments to prepare plans that fit capital requirements, hence ultimately draining the roles played by the local governments [8]. Ref. [72] calls this phenomenon ‘privatization of planning’ where the private sector lobbies corrupt officials to access land unchecked and uncontrolled by plans. Showcasing the Indian special economic zones (SEZ) practice. Ref. [73] identified how the SEZs law and practice in the country circumvent local tier governments not intervening in the SEZ territories using local and regional planning tools. Ref. [74] states that neo-liberalism introduced such ‘privatization of planning’ as widely observed in the planning and implementation of special economic zones in the global south.

**Fig. 1:**
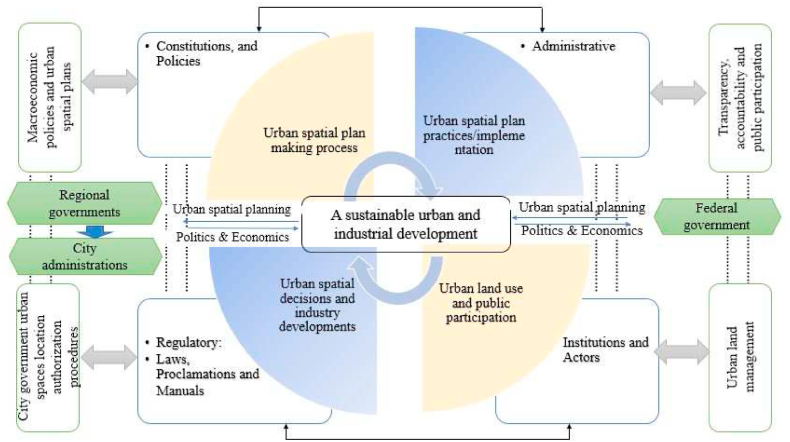
A conceptual framework to measure the performance of urban spatial planning practices in terms of industrial development.

Urban governance is taking a new shape in the 21st century as local governments globally are getting more leverage through decentralization efforts. Developing nations and, notably, African countries are evincing a leapfrog towards adopting decentralization policies mainly to respond to the needs of their rising population. Several factors drive decentralization, including political, economic, and, socio-cultural, and yet, it could not be a panacea for urban development and governance-related issues due to numerous reasons [75,76]. The juxtaposition of centralization - decentralization of urban planning practice in the global south indeed calls for further scholarly debate. From a benevolent political frontier, decentralization could help close to the grassroots, listen to the people, and act accordingly to avail better services and infrastructure. On the contrary, it could lead to misallocation and utilization of scarce resources, bad governance, and poor service delivery which always calls for a balance to the swing. Urban spatial plans and development regulations face strong public reactions and can quickly become highly politicized [34,67]. This has the power to capture why the preparation of the integrated master plan of Addis Ababa has failed [23]. Based on the interconnectedness of the selected variables wherein urban spatial plan-making and implementation process as a factor for sustainable urban and industrial development, and considering institutionalist and political economy perspectives, the subsequent framework has been developed for this study (see Fig. 1).

In sum, cities require better-performing industrialization for sustainable urban and industrial development, and industrialization requires better-functioning cities [77]. We also acknowledged that the Government of Ethiopia (GoE) calls for cities to guide their development according to spatial development plans [46]. The industry sector is expected to play a key role in the Ethiopian economy [48]. When discussing Ethiopia's industrialization landscape, it is a critical question whether the key institutions can carry out a policy or whether the people who staff the bureaucracies at the national and regional-state levels are technocrats hired based on meritocracy [78].

### 2.2 Urban spatial planning practices in Ethiopia

Ethiopia has a long history of urban spatial planning. The existing heritage sites in Yeha, Axum, Lalibela, Harer, and Gonder date back thousands of years and demonstrated the presence of some form of spatial planning exercise. The settlement around the present-day palace in Addis Ababa, established by Empress Taitu, is assumed to be the first guided settlement in the country [24]. Nonetheless, no guidance until the Italian occupation (1936–41) brought remarkable physical changes to the capital, Addis Ababa [79]. Foreign planners have strongly influenced Addis Ababa, most of whom failed to consider the local economic and social conditions [24]. This goes in line with the argument forwarded by Ref. [58] and, Ref. [68] who criticized planning systems in the global south for either inheriting planning systems of their previous colonial masters or wrongly adopting them from northern contexts.

In Ethiopia, proclamation No. 315/1979 urban planning law was the first to legalize urban planning issues. It defines the preparation and the content to be incorporated into urban planning. Recently, proclamation no. 574/2008 was enacted to establish a legal framework to promote planned and well-developed urban centers, and to regulate and facilitate development activities in urban centers, thereby enhancing the country's economic development [80]. The basic principles indicated in the proclamation include the capability of being implemented; ensuring public participation, transparency and accountability; and sustainable development [80]. Similarly, the recent urban plan preparation and implementation manual aimed at having capable planning and plan implementation institutions at different levels of government, bringing transparent and participatory planning and ensuring efficient and effective plan preparation and implementation system [50]. Within the framework of decentralization initiatives, efforts have been made to institutionalize decentralized urban government in Ethiopian cities. However, under the principle of ‘democratic centralism,’ regional and local structures control government institutions and remain loyal to decisions passed by the center [81].

Institutionally, despite the poor quality of plans and enforcement limitations, several urban centers in Ethiopia were able to have plans with the establishment of the National Urban Planning Institute (NUPI) in 1987, re-established in 2005 and was also dissolved later in 2008 with all of its powers transferred to the Ministry of Urban and Infrastructure (MoUDI). MoUDI designed policies, strategies, and implementation manuals and guidelines. After the controversial national election in 2005, the GoE shifted its focus from rural to urban areas [81]. Hence, The national urban development policy was developed and approved by the federal council of ministers in march 2005, which can be considered a shift in focus toward urban centers [43]. The national urban development policy aspires the urban centers to be industrial hubs by promoting agricultural and other modern manufacturing industries. To this end, the policy promotes the provision of land and infrastructure for industries [48]. The Federal Democratic Republic of Ethiopia (FDRE)^1^ constitution has stated many fundamental rights connected to urban development. Sub-articles under article 43 state three core concepts; the right to improved living standards, sustainable development, and the right to public participation [82]. Four-tier decentralization framework recognized in the Ethiopian context consists of regions (or states), zones (clusters of districts), woredas (or districts), and kebeles (or neighborhoods) [8,81,82]. City administrations are further subdivided into kebele administrations found at the lowest level of governance, and there is an overlap among the significant competencies between the structures [61].

**Fig. 2:**
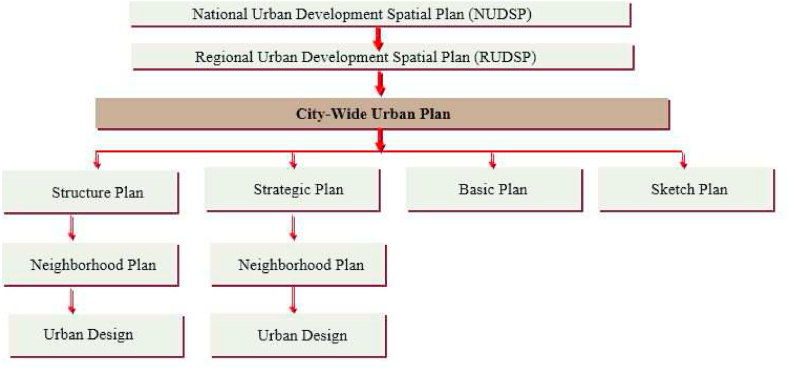
A framework of a hierarchy of plans in the Ethiopian urban planning system adopted from Ref. [50].

Ethiopia's most recognized planning approaches are structure, strategic, basic, sketch, neighborhood development plans, and urban design. These plans have been applied depending on the hierarchical stratification of urban centers. Hence, three hierarchies of plans in Ethiopia are recognized: national urban development scheme, regional urban development plan, and city-wide urban plans [50] (see Fig. 2).

A *structure plan* is a 10-year framework that guides land development in an urban setting and applies to level-one urban centers. It is a legally binding plan, along with its explanatory texts framed at the level of the entire urban boundary [80]. The structure plan preparation and implementation manual determined that urban land allocation for cities should be based on the 30-30-40 ratio for road and other infrastructure, greenery and open spaces, and residences and administration, respectively [50]. The terms ‘structure plans’ and ‘strategic plans’ are closely related [6]. These case cities adopted the structure plan approach, and OUPI prepared their structure plan in 2017 which is planned to last until 2027. As per Ref. [47] manual, each city with a population of 20,000 and over is autonomous to prepare its plan. However, most of the time, plans are conducted by highly centralized institutions like OUPI.

The structure plan shall have an implementation scheme comprising the institutional setup, resources, and legal framework [80]. Structure, master, or comprehensive plans give regulatory and political strength to spatial strategies [83]. Ref. [23] pointed out that be it a master plan, an integrated plan, a regional plan, or a structural plan, they all ought to be conceptualized in Ethiopia's social, political, economic, and environmental context. Similarly, Ref. [24] argued that planning as a state intervention tool needs to be conceptualized in the country context. Oromia regional state, one of the 11th regions in the Ethiopian federation, established OUPI by regulation No 67/2006 and re-established it several times till the recent restructuring (Proclamation No 242/2021). OUPI is an autonomous agency with all the policy initiation, regulation and supervision, and executive powers regarding urban plans. Thus, it is not only mandated with the preparation of different plans for urban centers and follow up its implementation but also prepare guidelines for the preparation of the same, and advise local governments. Until 2022, OUPI prepared approximately 152 structure plans for regional urban centers.

## 3 Methods and materials

### Ethical approval

Addis Ababa University (AAU) Senate Legislation of 2019 article 126 (3) clearly states that any research undertaking shall follow the rules and procedures of research standards, codes of professional ethics, norms, and responsibilities [84]. The Ethiopian Institute of Architecture, Building Construction, and City Development (EiABC), the Research and Technology Transfer Directorate (authors' affiliation), permitted the authors to perform the expert survey and field study in Galan and Dukem, city administrations. Till now, the EiABC has not yet set up a research ethics committee. However, we have contacted all participants with a support letter from EiABC, and, all survey respondents, including key informants, gave their informed consent. We explained the research's goal, ensured data confidentiality, and requested respondents' consent to be quoted correctly. This aided in gaining the trust and confidence of all participants, removing the anxiety of providing detailed information, and allowing respondents to freely share their experiences. The research participants were chosen voluntarily, meaning that they were not compelled to take part in the study. During a survey, key informant interviews, and field observations, the authors did not acquire any data without their approval.

### 3.1 Study area

**Fig. 3:**
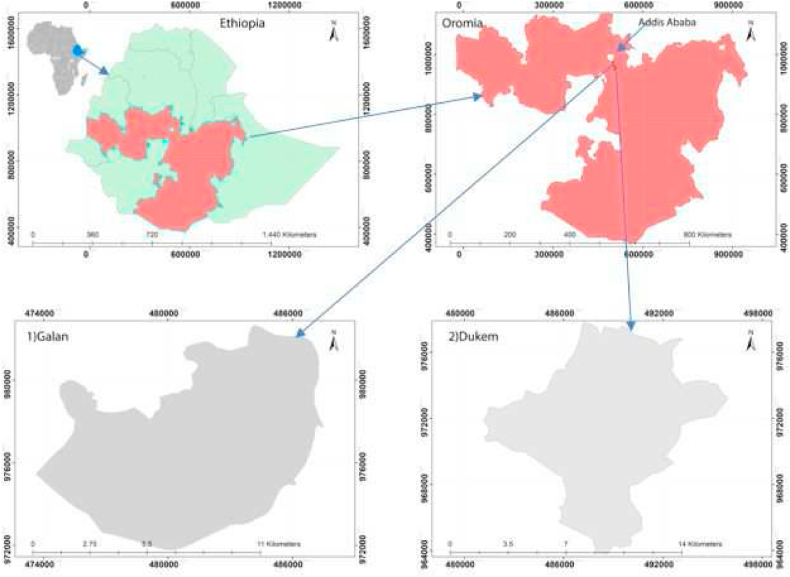
Location of study areas from Ethiopia and Oromia region perspective.

Galan and Dukem, are located in the Oromia Regional State 25 and 37 km south of Addis Ababa, respectively (see Fig. 3). These cities were selected for this study because (1) they are host to many (Table 1) investment activities and are facing rapid industrialization, (2) they are found next to each other and use a spatial urban plan prepared by the same institution (OUPI) [44,45], (3) urban land use plan violation and poor urban spatial planning implementation has been a common practice in the spatial units [18,20,29]. Astronomically, Galan and Dukem are located in an approximate geographical coordinate of between 8° 53′N – 8° 45′N latitudes and 38°46′E − 38° 56′E longitudes.

**Table 1:** Population, household, area coverage, and number of registered industries in the study cities.

Study cities	Total population size	Number of households	Area (in ha)	Operational industrial plants
Galan	76,800	21,333	9068	110
Dukem	85,839	23,884	9630	264

The cities are located along the highway between Addis Ababa and Djibouti. Information about population size, number of households, area, and the number of registered industries in the study cities are shown in Table 1.

The study cities are based on their level of development and population size are growing swiftly and fronting immense urban development challenges [8,30].

### 3.2 Data collection

The study was based on both primary and secondary data. Primary data were collected using a structured questionnaire from 69 experts in September/2022 and interviews with 21 key informants between May and November 2022 (see Table 2).

**Table 2:** Categories of respondents.

Respondents		Key Informant Interviews (KII)	Questionnaire Survey
Federal Ministry (MoUDI) officials, team leaders, and experts		3	15
Regional Institute (OUPI) officials and experts		3	15
City Administrations officials and experts	Galan	2	12
	Dukem	2	12
Industry developers	Galan	2	–
	Dukem	2	–
Residents	Galan	2	–
	Dukem	2	–
Consultants		3	15
**Total**		**21**	**69**

The primary reasons behind the selection of the above respondents in the expert survey include: both city administrations (Galan and Dukem) are responsible for implementing spatial plans. OUPI is an autonomous agency with all the policy initiation, regulation, supervision, and executive powers regarding urban plans in the Oromia region. The MoUDI is mandated to coordinate the preparation of a national integrated master plan and follow up on its implementation. The consultants were purposively selected as they know the ongoing developments in the two cities. Informal interviews with local communities were also made. Interviews with planners and other stakeholders also helped the authors to understand the intricate processes in plan-implementation activities' in the localities [13]. Institutional subjects were met experienced in their respective institutions, and selection was made purposively considering their involvement in planning and total job experiences. A similar approach was recently employed by Refs. [18,21,25,36]. Self-administered questionnaire links were sent to the survey participants through KoboCollect, a web-based application, where the interviewees completed and returned the filled questionnaires. A Likert-type question employing five scales (5 for strongly agree and 1 for strongly disagree) was used to gauge experts' perceptions.

Major questions raised to the key informants (KIs) include urban spatial planning decisions, structure plans making and implementation process, industrial developments trend, urban land use violations, level of public participation in spatial plan making and implementation process, and implementation capacity of cities during the spatial plan preparation. Physical observations in the two study sites were also conducted during the KIs sessions. Interviews lasted, on average, approximately 50 min. Questionnaires on the role of institutions and urban land use and urban spatial planning implementation process were adopted from the structure plan manuals [6,50]. The respective structure plan of the two cities (conducted in 2017), economic and physical plans, manuals, standards, directives, and national plans and policies were reviewed and analyzed as has been applied by Refs. [13,18].

### 3.3 Data analysis

**Fig. 4:**
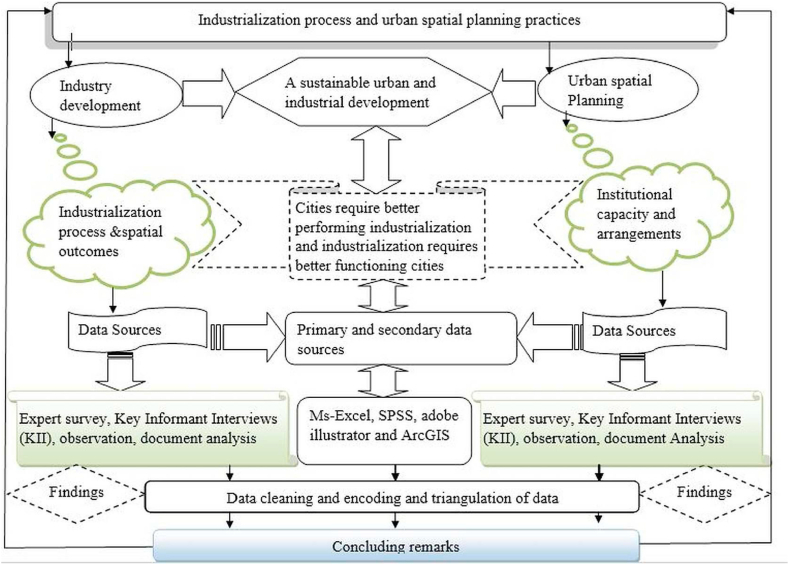
Methodological flow chart.

Cronbach's alpha reliability coefficient was used to verify data consistency and accuracy [85,86]. It usually ranges between 0 and 1 and if Cronbach's alpha coefficient is greater than or equal to 0.70, it is considered satisfactory [85,87]. Promax rotations were employed as correlation exists among the selected factors in the EFA analysis. The correction result indicates that the range of the reliability coefficient lies between 0.708 and 0.826, which is within the acceptable range (see Table 5). The procedures used for conducting factor analysis were: 1) identifying variables that are indicators of the performance of urban spatial planning practices, 2) conducting correlation analysis, also to justify factor analysis, 3) testing the general validity using the Kaiser-MeyerOlkin (KMO) and Bartlett's test methods, 4) applying Anti-Image Matrices Measure test, and finally, conducting factor analysis. SPSS software version 26.0 was used for data analysis [88,89]. All spatial analysis and Google Earth images were performed using a geographical information system (GIS) and Adobe Illustrator (see Fig. 4). Measures of central tendency such as mean and standard deviation were applied to display the number and percentage of household respondents for each variable.

**Table 3:** List of Likert scale type variables considered for the study.

Latent Variables	Variables code	Description of the variables
Institutions and actors' Involvement	UIS1	Urban institutions and land management are efficient.
	UIS2	Urban land management and land supply for industrial development are in line with the urban spatial plan.
	UIS3	Institutional enforcement capacity of government: in terms of professional human resources, is satisfactory.
	UIS4	Institutional enforcement capacity of government: in terms of protecting land use plans is satisfactory.
	UIS5	All actors are fully involved in developing urban plans (structure plans, local development plans, etc.)
	UIS6	There is strong vertical and horizontal integration in the city in implementing the envisaged plan with sector offices.
	UIS7	Rampant corruption aggravates land-use violations for industry development.
	UIS8	The objectives, targets, and strategies in the spatial plan implementation process are set clearly.
	UIS9	The legal and institutional framework provides enough clearness and transparency regarding urban land use and industry development.
	UIS10	Inefficient bureaucracies aggravate land use violations for industry development.
	UIS11	City government authorization procedures & standards in terms of land provision for industry development are clear.
	UIS12	There are loopholes in controlling land-use violations for industry development.
	UIS13	There is a lack of pertinent stakeholder participation in the urban plan-making process.
	UIS14	The lack of accountable local institutions contributed to haphazard development.
	UIS15	The existing local institutions that facilitate coordination between top political organs and the private sector are effective.
	UIS16	Urban spatial planning is challenging due to actors' competing interests (Government, communities, investors, activities, etc.)
Urban land use and the spatial planning implementation	IMP1	The level of urban spatial plan implementation is efficient.
	IMP2	There is urban spatial plan monitoring and evaluation.
	IMP3	There are a series of urban spatial plan regulations that are enforced.
	IMP4	Land use and management regulations are efficient.
	IMP5	A hierarchy of regional/detailed land use plans is specified by law.
	IMP6	Spatial plans guide urban spatial development.
	IMP7	There is a full implementation of the land use plans.
	IMP8	There needs to be more urban plan integration with neighboring cities/linkages.
	IMP9	Lack of land use policies contributed to haphazard growth.
	IMP10	The existence of unclear boundaries between cities makes plan implementation difficult.
	IMP11	Conflicting interests among actors contributed to poor implementation.
	IMP12	Lack of integration/linkages between cities makes plan implementation difficult.
	IMP13	A highly centralized urban spatial plan-making process contributed to the poor implementation of city plans.
	IMP14	The national and urban level frameworks undermine uncontrolled development.
	IMP15	The lack of an integrated approach to urban spatial planning leads to urban problems and poor plan implementation.
	IMP16	Poor urban land management practices contributed to a violation of urban spatial plans.
Level of public participation	PP1	A series of regulations are generally justified based on overall public interest.
	PP2	There is transparency in land-use restrictions. Changes in land-use and management regulations.
	PP3	Existing urban land use rights and change information is publicly available.
	PP4	There is citizen participation in urban planning practices.
	PP5	The community or representatives of different stakeholders participate in the supervision of the plan implementation process.
	PP6	There is transparent land delivery for industry developments.
	PP7	There is no access to know about what has been planned in cities.
	PP8	There is no way to incorporate a public hearing into the plan.
	PP9	There is participation in the industrial development process.
	PP10	There is limited stakeholders' participation in the industry policy-making process.
	PP11	There is transparency in land allocation for investments.

**Table 4:** Measures of central tendency, symmetry, and taildness for the study of latent variables.

Latent variables	Variables code	Mean	Skewness	Kurtosis	Standard Deviation (SD)
Institutions and actors' Involvement	UIS1	1.93	0.94	−293	1.05
	UIS2	2.17	0.57	−926	1.08
	UIS3	2.09	1.07	1.08	0.98
	UIS4	2.04	0.89	−052	0.97
Urban land use and the spatial planning implementation	IMP10	3.94	−1.84	4.55	0.98
	IMP11	3.97	−1.94	5.49	0.84
	IMP12	3.93	−1.57	3.36	0.94
	IMP15	4.25	−2.15	5.46	0.96
	IMP16	4.14	−1.66	3.26	0.92
Level of public participation	PP6	2.06	0.83	0.24	0.98
	PP9	2.14	0.99	0.38	1.10
	PP11	2.01	0.91	0.15	1.02

**Table 5:** Alpha results before and after the correction made.

Latent variable category	Total Cases	Without correction		With Correction		
		No.of variables	Alpha result	No.of variables	Alpha result	Remark
Institutions and actors' Involvement	69	16	0.651	12	0.708	4 Items were deleted (UIS13,14,15 and16)
Urban land use and the spatial planning implementation	69	16	0.535	11	0.717	5 Items were deleted (IMP1,2,3,4 and 5)
Level of public participation	69	11	0.669	8	0.826	3 Items were deleted (PP7,8 and 11)
Total		43		31

## 4 Results and discussion

### 4.1 Respondents' background information

Of the surveyed 69 experts, 65% were male, and 35% were female. All respondents fall in the age category of 30–65 years. About 55% have 8–15 years of work experience, and 45% have 16–65 years of work experience. Regarding their educational achievement, 3% of the respondents have a diploma, 28% have a first degree, 67% have a second degree, and 8% have a Ph.D. or above levels of education. The composition of the respondents’ expertise encompasses planners (30.4%) (n = 21), economists (18.8%) (n = 13), land management experts (8.7%) (n = 6), sociologists (7.2%) (n = 5), geographers (7.2%) (n = 5), architects (6%) (n = 4), civil engineers (5.8%) (n = 4), environmentalists (4.3%) (n = 3), urban managers (4.3%) (n = 3), and others (5). This indicates that the survey respondents include an optimum mix of professions required to undertake spatial planning [50]. The variables' descriptive statistics, including mean and standard deviation, were calculated (see Table 4). A Likert-type question employing five scales (5 for strongly agree and 1 for strongly disagree) was used to gauge experts' perceptions of the performance of urban spatial planning. Respondents were asked about their perception of institutions and actors' involvement in urban spatial decisions considering sustainable urban and industrial development.

**Fig. 5:**
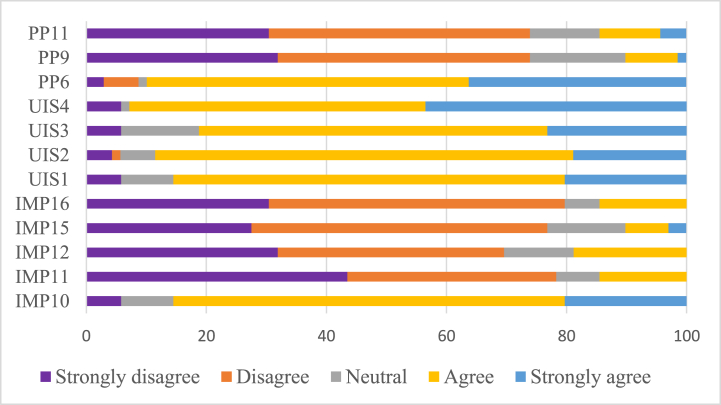
Results of factor analysis (%) for the applied latent variables.

About 78.3% (n = 54), 69.6% (n = 48), 76.8% (n = 53), and 79.7% (n = 55) disagreed or strongly disagreed with UIS1, UIS2, UIS3, and UIS 4, respectively. Whereas, about 85.5% (n = 59), 88.4% (n = 61), 81.2% (n = 56), 92.8% (n = 64) and 89.8% (n = 62) agreed or strongly agreed to IMP10, IMP11, IMP12, IMP15, and IMP16. About 73.9% (n = 51), 73.9% (n = 51), and 75.4% (n = 52) disagree or strongly disagree with PP6, PP9, and PP10 (see Fig. 5). This implies that most experts are unsatisfied with institutions' efficiency and performance in urban spatial planning practices (UIS1, UIS2, UIS3, and UIS4-latent variable institutions and actors' involvement indicators). Likewise, most experts indicated that there is weak urban spatial planning implementation performance as shown under IMP 10, IMP11, IMP12, IMP15, and IMP16. The third latent variable is the level of public participation, measured by variables PP6, PP9, and PP10. The result depicted that most experts perceived the absence of public participation in the industrial development process.

### 4.2 Exploratory Factor Analysis (EFA) results

As listed in Table 3, 43 Likert scale-type subjective items were considered for the EFA to examine the performances of urban spatial planning factors. Variables that score low in Cronbach alpha results were deleted. Hence, 4 items from UIS (UIS13,14,15 and 16), 5 items from IMP (IMP1,2,3,4 and 5), and 3 Items from PP (PP7,8 and 11) were deleted, and a minimum Cronbach alpha threshold of 0.7 achieved (see Table 5). Consequently, the number of variables considered for the EFA analysis was reduced to 31.

Factor coefficients with a minimum value of 0.5 were considered acceptable in the factor construct. Factor cross-loadings were also rejected to make the construct simple and robust. Each construct with three acceptable elements, Cronbach alpha reliability test result above 0.7, and theoretical soundness was considered to present the final solution, which passed six successive tests. The process required removing problematic items with low loadings (below 0.5). The final KMO result obtained was 0.728 with 12 urban spatial planning performance indicators that load under three dimensions have significant Bartlett's Test of Sphericity (χ2 = 518.198, p < 0.000). As a result, all factors in this factor analysis can be further processed [90]. One commonly used technique is Kaiser's criterion or the eigenvalue rule. Under this rule, only those factors with an eigenvalue of 1.0 or more are retained [91,92]. Using this criterion, the dimension is reduced to 12 variables explaining 75% of the variability (Table 6).

**Table 6:** Factor Loading Matrix for the reduction of performance of urban spatial planning indicator.

Urban spatial planning performance indicators	Factors			Cronbach alpha
	1	2	3	
IMP10	0.939			0.867
IMP11	0.848			
IMP12	0.837			
IMP15	0.822			
IMP16	0.765			
UIS1		0.873		0.922
UIS2		0.805		
UIS3		0.778		
UIS4		0.708		
PP6			0.847	0.797
PP9			0.782	
PP11			0.636	
Eigenvalue	4.179	2.902	1.922	
Percentage of variance Explained	34.829	24.181	16.017	
Total variation explained	75.028			
Extraction Method: Principal Axis Factoring.				
Rotation Method: Promax with Kaiser Normalization.				

The reliability test of the items, as indicated by Cronbach alpha, reveals that the above items are reliable [87,93]. Thus, these items could serve as a measure of a latent variable of this study.

#### 4.2.1 Perceptions of respondents on urban spatial planning performance

The first indicator of urban spatial planning performance (USPP) is institutions' and actors' involvement in urban spatial decisions. Respondents were asked about their perception of urban institutions and land management efficiency, urban land management and land supply for industrial development, and the institutional enforcement capacity of the government in terms of professional staffing and institutional enforcement capacity in terms of protecting land use plans. Hence, respondents’ scores with a mean value of (1.9), (2.2), (2.1), and (2.0) respectively suggest that the institutions' and actors' involvement in urban spatial decisions are poorly considered in the urban spatial plan. In contrast, the institutions and actors' involvement in urban spatial decisions is important in urban spatial planning decision-making. As per Ref. [61], strengthening urban institutions at present will be essential for healthy urbanization in Ethiopia, where the urbanization level is low. Some of the institutional inefficiencies emanate from administrative problems and staffing quality. A MoUDI interviewed expert attests that “*Inadequate and unqualified staffing during the plan-making process and implementation phases contribute to land use violations and create loopholes for corruption. Planners, local leaders, and land-related experts should be trained and get continuous on and off-job training*”. The quality of planning depends on how planners are trained [18], which also enhances proper urban structure plan implementation [36]. A practical and well-trained professional civil servant is a crucial component of policy execution [78].

Among other things, the limited capacity of urban and regional planners, municipal urban designers, and related expertise partly contributed to a haphazard urban settlement that has also been aggravated by migrants looking for economic opportunities. Consultation and collaboration are indispensable for successful spatial planning [15,55]. Cities are a product of the interaction between political and economic decisions made by several actors [24]. The urban spatial plan-making and implementation process naturally involves many actors and decisions, often interdependent. Moreover, institutions are crucial in contexts where many interdependent decisions and agreements occur [51]. The creation of urban spaces is a result of an imbalanced interaction among global, national, and municipal actors, each with conflicting aims [15]. Pertinent stakeholders' participation and institutional collaboration are indispensable for the effective implementation of urban spatial plans [25,36]. A senior policy consultant pointed out “I*nadequate planning, haphazard developments, and unsustainable use of land and natural resources can increase risk. The complex spatial relationship between industrial developments, their host cities, and surrounding settlements must be tackled through appropriate urban spatial planning and instruments*”. This is similar to what is stated in Ref. [18]. According to Ref. [18], the institutional framework determines the effectiveness and implementable spatial planning in which it is anchored. Ref. [2] also pointed out that deficiencies in spatial plan implementation are an issue of governance. A study by Ref. [94], attests that national urban policies in Sub-Saharan Africa largely seem unfeasible due to a lack of “technical capabilities, legal frameworks, financial instruments, and political will” to effectively realize policy elements engraved.

The second urban spatial planning performance (USPP) indicator is urban land use and spatial planning implementation. Respondents were asked about their perception of implementation-related problems on the existence of unclear boundaries between cities, conflicting interests among actors, lack of linkages between cities, an integrated approach to urban spatial planning, and poor urban land management practices. The score in this case has a mean value of (3.94), (3.97), (3.93), (4.25), and (4.14), respectively (see Table 4). Urban plan implementation stage is the hardest part of the urban spatial planning process in Ethiopia [36] and plan implementation evaluation is difficult in fast-growing cities [38]. Weak urban spatial planning systems must implement approved policies effectively [4]. An official working in Dukem city administration explains that “*Urban spatial planning implementation has been facing severe institutional challenges, including a lack of experts. There have been serious land use violations. Above all, the fact that OUPI is making plans for Oromia urban centers shows this city's lack of capacity/institutional inefficiency. In addition, the plan is highly top-down”*. The government should ensure that steers the development as per plan and avoid violations without a convincing reason based on public interests [27]. Yet, in Ethiopia cities fail to implement their structure plan within the specific implementation phase [36].

The third urban spatial planning performance (USPP) indicator is public participation in urban spatial plan-making and implementation. Respondents were asked about the transparency of land delivery for industry developments, participation in the industrial development process, and transparency in land allocation for investments, and (2.06), (2.14), and (2.01) mean scores were recorded in these regards. This implies the need for more public participation and transparency in the industrialization development process. A consultant in the area also explained, “*Local governance and urban management are weak and lack effective citizen participation despite the presence of various development committees at the grassroots formed to ensure accountability or proper financial management*”. The outcome is consistent with Ref. [8]'s findings which demonstrate low participation patterns while establishing special economic zones in Dukem and Addis Ababa cities.

In the Galan structure plan preparation process concerning community participation, the Galan city administration resident explains that “*There were no serious public discussions and input gatherings during the plan preparation process*”. Similarly, a consultant explained, “*Urban plans should be prepared and carried out in an open, accountable, and participatory manner. However, public engagement and local community ownership of plans are severely constrained due to a failure to consider public knowledge and involve them in the process of plan making and implementation*”. Art.43.2, Art. 89.6 Art 92.3 of the FDRE constitution stipulates the need to ensure the participation of people in the overall national, economic, and environmental policies and projects [82]. This includes the public right to know the details of any development activities during the designing and implementation process. To this end, various institutions are established at different hierarchies including, the House of Representatives at the federal level, the Regional Council at the regional level, and the *wored*a council at the *woreda* level. These institutions have not so far ensured community participation as it is participation by representation and is more centrally manipulated. There ought to be greater public participation, including the right to identify the problem and get information concerning the plan [6,19,23,36]. All-inclusiveness has yet to be given substantial consideration in global south planning practices [58].

Urban spatial planning practices not based on participatory approaches are evident in weak planning [4]. All key informant interviewees from Galan and Dukem administrations are critical of the transparency of the plan contents. The structure plan guides urban development activities as a whole, and its contents shall be displayed to the public for at least 2 weeks before approval and implementation [47]. OUPI experts argue that “*They engage the public in the plan-making process, albeit they underlined that the level and continuity of public engagement need more depth*”. Public participation is a decision-making process [35] and helps to prevent spatial conflicts and to implement the plan to the expected level [19,36].

Based on an EFA (Table 6), the KMO measure was 0.728, and Bartlett's Test of Sphericity ×2 = 518.198 (df) = 66, p < 0.000 indicating that the data were likely factorable. The three factors were extracted, representing 34.8%, 24.1%, 16.1%, and 75% of the total variances. Five items were loaded highly in the first factor. These are IMP10, IMP11, IMP12, IMP15, and IMP16 loaded with (0.939), (0.848), (0.837), (0.822), and (0.765), respectively. This implies the importance of urban land use and spatial planning implementation on the performances of urban spatial planning. The second factors are UIS1, UIS2, UIS3, and UIS4 loaded with (0.873), (0.805), (0.778), and (0.708), respectively. This implies that the importance of institutions is indispensable for a successful urban spatial plan implementation. The third factor is PP6, PP9, and PP11 loaded with (0.847), (0.782), and (0.636), respectively. This shows the role of public participation in the plan-making and implementation process. Planning is an interactive process undertaken in a social context rather than a purely technical process [55]. Ref. [95] contends that all stakeholders should be included in collaborative processes that result in planning decisions and that these processes should adhere to specific norms that guarantee participation is equitable, equal, and empowering. Ref. [25] also calls for vertical and horizontal collaboration among stakeholders in urban spatial planning practices.

In a perfect spatial planning process, all the above elements must be well coordinated [21]. Careful and far-sighted planning is needed to secure progress from urbanization and minimize the threats of urbanization [40]. Overall planning for space and land is the essence of city management [2,6], and cities have been planned in one way or another, in the broadest sense of land and space management [55]. Countries with weak urban spatial planning institutions are characterized by insufficient human and poor institutional capacities [4]. One way out is to have plan-led industrial and urban development using spatial planning tools [43]. In Ethiopia regulatory, policy, and institutional frameworks have been crafted over the past two decades to strengthen the manufacturing sector [48,[96], [97], [98]]. Thus, more than having a planning agency and an effective bureaucracy is required to bring about industrial transformation [78]. The process of industrialization should be transparent and implemented to keep decision-makers accountable and benefit society rather than just a few groups [11]. Addis Ababa is the capital and primate city, and through “poly-centric development” forming a strong linkage with the surrounding cities is indispensable [48].

### 4.3 Urban spatial plan-making process and implementation

Considering urban spatial planning as a process is as important as the objectives and contents of the plan. This implies that how and where to locate industries should not be the only main aim of planning but also the planning process, how issues are discussed, and how problems are defined are necessary [55]. The implementation gap is a crucial problem in the planning process [2,36].

**Fig. 6:**
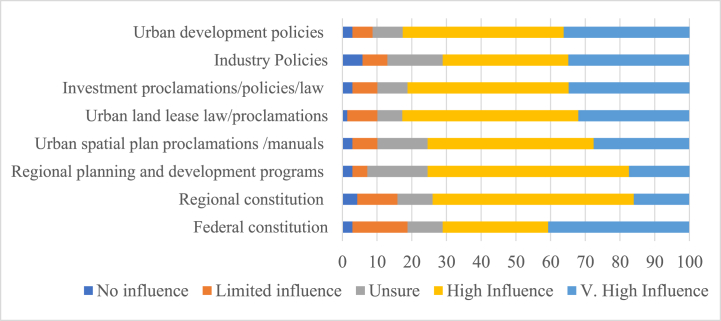
The influence of major laws and policies on the urban spatial-making process in Ethiopia (%).

**Fig. 7:**
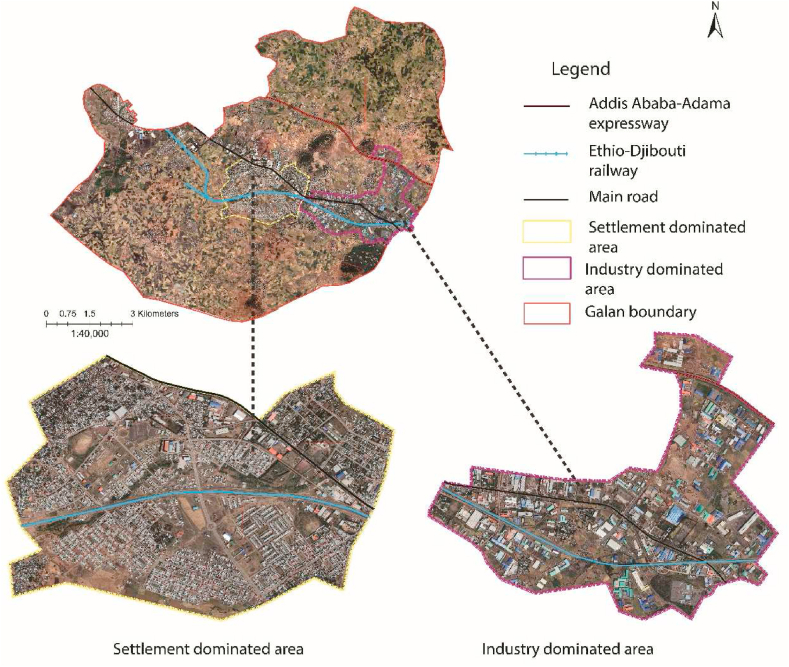
Google earth pro image showing settlement and industry-dominated areas in Galan (2022).

Key informants in the cities claim that “*Policies, directions, and urban plan guidelines do not timely reach their administration. Institutional instability has been a serious problem and resulted in poor implementation of plans. Often senior experts reshuffling made and mayors change frequently*”. Ethiopia's previous development plan served for 2015–2020 highlights the costs of uncoordinated urban development and calls for strategic urban spatial planning [46]. The respondents were asked to gauge the impact of some variables that can influence the urban spatial plan-making process in the context of industrializing cities. Accordingly, 71%, 64%, 66%, 84%, and 82% of the experts perceived that federal constitution, regional constitutions, regional planning programs, urban spatial plan proclamations, and urban land lease law have a powerful influence on urban spatial plan-making process respectively (see Fig. 6) (see Fig. 7).

In addition, a consultant explained that “*There is no consideration of space in industrial policies and lacks attention during urban spatial planning preparation*”. Ref. [1] explains that the capacity of a planning system to integrate industrial development is limited by the power of sectoral policy communities and the weakness of the conceptions of sociospatial relations. The quality of spatial planning depends on the policy framework in which planning goals are developed [18].

**Fig. 8:**
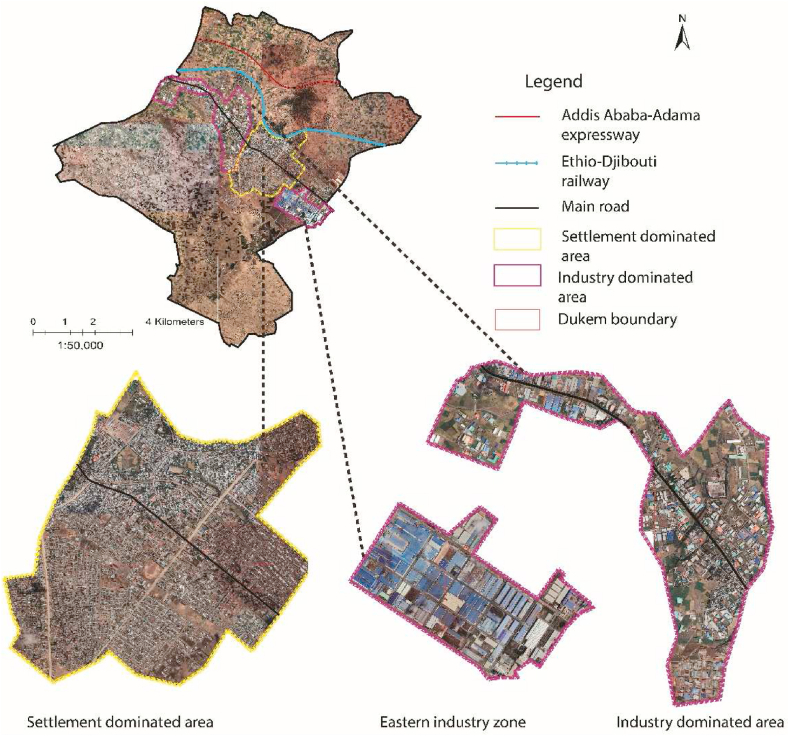
Google earth pro image showing settlement and industry-dominated areas in Dukem (2022).

Ethiopia's manufacturing sector has been among the key productive sectors of the economy identified by the government [96,98,99]. As per the structure plan of the case cities, 110 and 264 operational industrial enterprises occupied 625 ha, and 540 ha, of land in Galan, and Dukem, respectively [44,45] (see Table 7, Table 8). Land allocated for industries is the most underutilized compared with other land uses in the surrounding cities of Addis Ababa [12]. In 2017, OUPI prepared a new structure plan for Galan City, including a land use plan. Hence, a considerable gap has been observed in the manufacturing proposal, which shows 19% against 5–10% of the manual standard (see Table 7). OUPI expert explains that “*The shortage of qualified urban spatial planning experts was the leading challenge they encountered during the preparation of Galan and Dukem structure plans in 2017*”. Likewise, OUPI expert explains that “Planners and architects mainly undertake planning*, and all other remaining disciplines either do not participate at all or are considered and employed as* supporting *staff*”. Engineers' and architects' domination of the planning activity in the urban physical planning-making process ought to be changed [55,83] (see Fig. 8).

**Table 7:** Proposed land use and implementation performance of Galan City (2008–2017).

LULC	Proposedland use (ha)	%	Implementations (ha)	Standard (%)	The gap from a standard (%)
Social services	733.5	9.7	125.4	5–10	Within range
Manufacturing	2122.1	28.3	677.5	5–10	+19
Commerce	238.5	3.2	32.7	5–10	−1.8
Green area (Park, recreation, and open spaces)	1086.4	14.5	6.0	15–20	Within range
Reserved area	975.4	13.0	–	–	–
Residences and administration	1259.6	16.8	285.3	55–70	−38.4
Infrastructure	1100.6	14.6	–	15–25	−0.36
Total	7516a	100			

a × the remaining land is proposed and allocated for other land use classes.

Key informants from OUPI confirmed that land use land cover violations were severe problems in the city administrations. Key informants from the Galan city administration also share the same view as those from OUPI. According to these informants, the federal government also changed land use in the name of investment expansion. There needs to be accountability in how officials allocate land for industrial development with no far-sighted plans. Interviewees from Dukem city administration explained that: “*There has been a violation of the city plan that abused the city's land-use plan. This impeded the city's development since integrating planned settlements with unplanned settlements built on public land is challenging*”. The discussants from both Galan and Dukem also explained: “*The industrial development that is uncoordinated and dispersed has hampered coordinated development in each city*. “Strategic work” is needed for urban areas designated for industrial development [1]. Ethiopia's prior policies before the 1980s seemed urban-biased through the nationalization of urban land [41] and then rural-biased in the 1990 ^th^ under the ADLI strategy after the fall of the Derg regime (1974–1991) [96,97]. One missing issue in the discourses of urban planning in developing countries is the impact of “ideological swings” which affect urban spatial planning [34].

### 4.4 The spatial distribution of the existing industries, urban expansion, and land allocation

There has been a lack of effective integration between policies concerning space organization, land allocation, and industrial development [100], and Ethiopian industrial policies remain space-blind [30]. It takes work to get reliable data on land delivery for investments. An interview with a consultant attests that " *There is a deep level of poor documentation on land delivery and difficult to trust any data on land allocation. Poor and inappropriate documentation of land allocation is exposed for manipulation. City land administration partly hides such information because the land has been delivered largely corruptly. Similarly, plans are often considered a classified document and only a few can access it*”. OUPI expert also claims that “*The lack of up-to-date data and data management systems has been a severe problem in plan preparation*”. The mandates on land allocation and administration to the different levels of government within a regional state are usually determined by unpublished administrative directives and without public notice [11]. These practices are not in line with the principles of good governance. OUPI expert explains that “*Interference of regional and federal government officials in plan preparation and implementation resulted in serious violation of planning principles and land use violation that often change agricultural lands and public spaces to commercial or industrial uses*”. When there is a violation of plans and is proven by supervision, the concerned higher authority is responsible/accountable for it [47].

Both legal and illegal actions by various actors resulted in land development [5]. Land is a very contested resource in Ethiopia and unambiguous: in terms of land transfer or allocation issues [11]. Hence, the FDRE constitution Art 40 (3) clearly states that the right to ownership of rural and urban land, and all-natural resources, is exclusively vested in the State and the Peoples of Ethiopia [82]. Following Derg's downfall in 1991, the EPRDF introduced a leasehold form of the urban land system to the country. Indeed, land remained under government control since 1974, although the rights to use the resource and its improvements varied during the Derg and the incumbent regimes [101,102].

An industrial developer in Galan City indicated, “The land lease policy states that in cases where regional governments receive an application for the allocation of land for an approved investment, they shall deliver the required land to the investor within 60 days. However, when it comes to reality in the city, acquiring land through the lease-holding system has been pronounced by many as time-consuming. And investors claim to have been discouraged by the long bureaucracy of handling the land lease system”. The development of Galan and Dukem cities depends on the manufacturing sector's performance. Yet, macro-level policies and investment procedures connected to space organization and industrial development must be smoothly integrated [6]. As one of the policy measures, the Ethiopian government had been taking the urban land lease mechanism to enhance the transfer of land use rights, value the urban land and encourage investment and proffer social services to the residents [103].

**Fig. 9:**
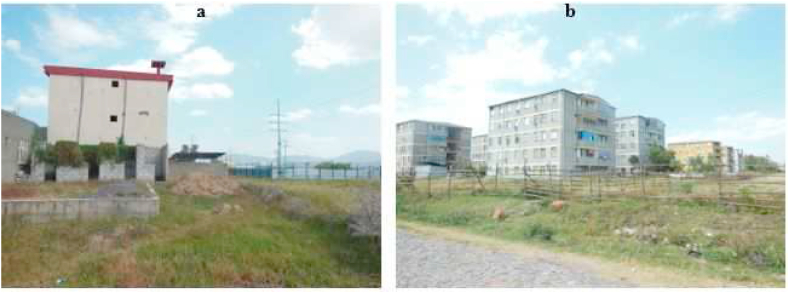
Private residential (a) and government condominium housing (b) areas near the Eastern industry zone in Dukem (2022).

The incompatible location of manufacturing industries within residential areas has been a serious concern for the residents *(see* Fig. 9*).* A resident in Dukem city attested, “*Industry-related services pose problems related to noise, air and water pollution, and traffic congestion. Due to the lack of proper land use regulations and partly unplanned city growth, these industries in the inner city pose health and other socio-economic problems to many residents*”. Planning is ethical as it raises queries about what should be done, for whom and by whom, and who benefits or losses [57]. Investment expansion is one of the contributing factors to urban land use changes and puts pressure on the urban spatial plan-making process [104]. Research by Ref. [78] gives solid empirical support to the positive effect of industrialization on local communities. Thus, more than having a planning agency and an effective bureaucracy is needed to bring about industrial transformation.

**Fig. 10:**
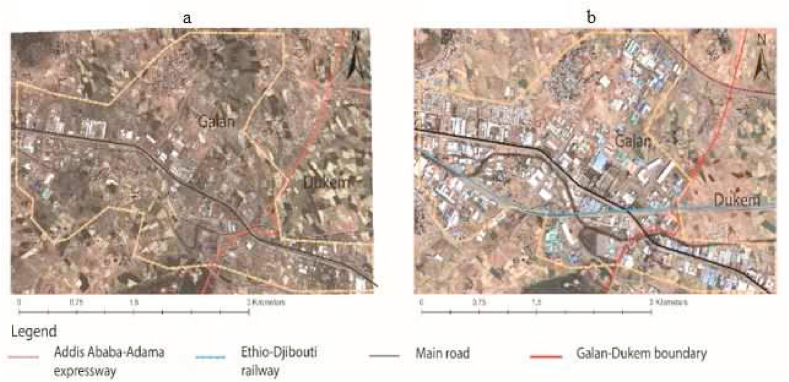
Industrial and built-up areas growth; Galan-Dukem in 2010 (a) and Galan-Dukem in 2022 (b); computed based on Google earth pro image.

Most cities initially expanded with a minimum government consideration agreed to plan in the expanding urban fringe [5]. As per the structure plan of each study city, approximately 40.12 ha and 98 ha of their land proposed for green areas have been invaded by informal settlements in Galan, and Dukem, respectively [44,45]. A better understanding of urban spatial expansion is indispensable for sustainable urban development [26]. Unruly urban expansion problems can be minimized by implementing structure plans appropriately [36], but spatial planning fails to regulate urban expansion [22]. There is no automatic link between rapid urban growth and urban problems, albeit, institutions matter most [5]. The processes of industrialization tend to be accompanied by spatial expansion, resulting in encroachments of agricultural land [28,29]. The prevailing practices of land management cause constrained land supply and spatially fragmented development [61]. Undoubtedly, the studied cities have had continuous industrial growth and fast urbanization in recent decades, which calls for the development of various types of infrastructure. (see Fig. 10).

### 4.5 Institutionalist and political economy approaches: a prima facie reasons in urban spatial plan-making and implementation irregularities

Experts perceived that federal and regional government intervention contributed to urban land use violations indicated by higher mean values, 3.99 and 3.77 respectively (Table 8). Galan and Dukem city residents supported this and pointed out that “C*ities mayors and their cabinet members violate urban land use plans and provide land for investors without any consultations*”. This is consistent with a study by Refs. [39,67] who noted that urban development is linked with political interference. According to Ref. [51], institutions are not just rules for making decisions but also define benefit distributions. To enhance coordination and collaboration in urban activities, it is imperative to rethink the institutional approach to urban interventions [62]. Ref. [55] depicts that spatial planning reveals much about a society's capacity for cooperation across diverse interests. In urban land allocation, the politicians' arm is stronger than the legal way. In this case, the power of the market forces is more triumphant than politics. More importantly, these case cities were located next to a primate city, Addis Ababa, which resulted in soaring urban land value. Recognizing the importance of urban governance and institutions is essential to correct maladministration in the process of urban spatial plan-making process and implementation [6]. A study by Ref. [33] demonstrated that the absence of superior laws resulted in many problems in China's spatial planning.

**Table 8:** Experts' perception of industrial and urban spatial planning sustainability issues.

Items	Likert scale					
	1	2	3	4	5	Mean
Federal government intervention in promoting industrial development resulted in a violation of urban land uses	0	6	12	28	23	3.99
Regional government intervention in promoting industrial development resulted in a violation of urban land uses	2	14	9	17	27	3.77
Cities' urban spatial plans guide the industrialization process	15	37	9	1	7	2.25
National industrial policies are space-blind/do not give due consideration to geography/location	0	6	13	30	20	3.93
The highly central urban spatial plan-making process is a bottleneck to sustainable industry and city development	0	9	8	20	32	4.09
There is a strong spatial relationship between industrial developers and host cities	24	26	7	10	2	2.13

An expert survey also shows that a highly centralized urban spatial plan-making process creates problems, indicated by a higher mean value of 4.09 (Table 8). As evidenced in this study, urban spatial plans of both Galan and Dukem cities were designed centrally by the institution called OUPI. A consultant explains that “*Planners designed and proposed industrial locations without knowing the ground fact. A powerful central command in urban spatial decision-making and the federal government using its party-affiliated regional government offices commanded cities to prepare land for manufacturing industries without prior notice and consultation with the city's administration*”. Planning actions are related to a good understanding of local conditions [6,33]. FDRE constitution gives autonomy to regional states, but its practicality has often been questioned [81]. Such unfortunate occurrences have created discrepancies in the plan-making and implementation process [2]. In contrast, one of the reasons why Kigali's political bargaining environment turned conducive to effective plan implementation was a highly centralized political system that streamlined relations between planners and mayors at the city level and the central authorities [34]. Winning decentralization features tackling challenges associated with unclear mandates, weak redistributive mechanisms, uneven service quality, and limited citizen participation [76].

Experts perceived that industrialization is not guided by cities' urban spatial plans, indicated by a mean value of 2.25 (Table 8). Urban spatial planning seems to fail in controlling urban sprawl due to several political and economic factors in the global south [13]. Industry developers stated that “*Locating manufacturing industries often were done with political intention and not with clear policy directions and pieces of knowledge. On top of this, local cadres violate land use regulations indicated in cities' spatial plans*”. Experts perceived that national industrial and macroeconomic policies are space-blind, indicated by a mean value of 3.93. Place-blind evolution in public policy is strikingly evident in highly centralized countries [1]. The space blindness of policymakers needs to be addressed [30]. On top of these, developing sector-specific plans is like working in a separate silo. Ref. [18] also pointed out that failure in urban spatial planning resulted in a drastic impact on the residents, and an all-rounded urban spatial planning role is indispensable.

According to the surveyed experts, there is a lack of spatial relationship between industrial developers and spatial plans of host cities, which is indicated by a mean value of 2.13 (Table 8). Ethiopia's urban planning and development issues are of constitutional concern. Therefore, more than in other countries, urban spatial decision-making process affairs require tremendous political will to fulfill the needs of the industrial developers. Ref. [55] recognized that planning exercises are not neutral in terms of values. Government actions in urban planning are therefore likely to have a variety of goals. Ref. [24] noted that government intervention in developing countries influences the pattern and morphology of cities. Evidence from the case cities indicates a striking too frequent pattern of changing mayors. Dukem and Galan city administrations confirmed, “*In the past two years, they knew at least 2 mayors each*”. The need to consider the dynamic nature of politics in developing countries in urban planning practices has been one of the limitations [2,34,67]. This may imply establishing functional institutions, staffing capable and accountable personnel, nurturing a benevolent political system, and building a robust political economy framework. It is not untrue that in developing countries like Ethiopia, where the democratic system is in its infancy, market forces do not function unimpeded and are considerably dictated by politics.

Political interference with city planners' activities affects the effectiveness of planning [34]. In the Ethiopian industrialization process experts appointed based on political party affiliation tend to conform to the party line and exhibit little independent thought [78]. Politicians rarely share a technical discourse concerning the strategic development of cities [4]. Another issue worth discussing relates to the urban plan implementation practice in Oromia region under the country's decentralization framework. Ethiopia officiated decentralization in its 1995 constitution. Art. 50 (1) of the constitution empowers regional states to self-administration in their geographic proper although cities are not mentioned as an independent structure similar to *woredas* of *kebeles* [105]. It seems OUPI draws its rights and actions based on the constitutional right bestowed to regions. However, the Ethiopian urban planning law (Procl. No. 574/2008) gives urban centers the right to prepare and implement urban plans by ensuring public participation, which is quite contrary to what the OUPI centralized planning exercise. Understandably, decentralization could not be a panacea for urban development and governance-related issues because its success depends on several factors [75,76]. Ref. [76] identifies a lack of institutional strength as a major bottleneck for the effective implementation of decentralization, especially in African countries. A study by Ref. [36] confirms that the Ethiopian urban plan implementation guideline is violated by local governments and lacks an institutional setup for plan implementation. As Ref. [34] highlighted, political economy analysis is essential when analyzing the effectiveness of urban spatial planning and development regulation.

## 5 Conclusions

The result of the study attests that industrial development in the studied cities should be implemented in line with urban spatial plans. The participation and transparency on urban spatial development issues by concerned stakeholders should be enhanced. The political decisions of the executives (all lower tiers of government structures and their cabinet) need to be institutionalized to implement urban spatial and industrial plans properly. To this end, all actors must understand the intents of urban spatial plans, policies, laws, and proclamations. The interaction between the structure plans of city officials and the unforeseen impacts of industrialization on the ground reveals the dynamic tension between land use and investment in the urban context. As evidenced in this study, politics, and economics strongly influence the urban spatial planning making and implementation practice under a highly centralized planning institute. Regardless of their different administration units and location, the urban spatial of both cities were prepared by a centralized institute called OUPI. This study argued that similar political bargaining environments resulted in similar urban spatial plan implementation performances in both cities. In both studied cities, urban spatial plans and industrial development are apolitical and aspatial, respectively. Alas, industry development takes place in physical space. This implies that industry locations must be organized considering spatial and socio-economic elements.

The right land allocation policy and implementations that can facilitate businesses to grow and prosper are indispensable. To fully capitalize on the potential of the study cities' available resources and fully implement the urban spatial planning proposals, the institutional capacity of cities needs to be strengthened. The federal, regional, and city-level offices, must aim the study cities' sustainable industrial and urban development. Furthermore, there must be a democratic framework that allows citizens to influence urban spatial plans. Thus, urban spatial planning activities that affect the interests of communities should consult the public. This research shows that cities lack crucial components for democratic governance, such as a public hearing platform. Besides, urban planners need deep and well-informed technical expertise in urban planning. In addition, since the case cities are found next to a primate city, Addis Ababa, and form a conurbation, an integrated regional development spatial plan has to be promoted in the area. However, we do not claim that integrated planning is a panacea for all urban spatial planning problems. There should be an industrialization process that considers the local urban spatial plan. Both must be designed to contribute to the sustainable development of urban centers, which in turn requires efficient regional, local, and federal institutions. If urbanization and rapid industrial development are not adequately planned and managed, their drawbacks may outweigh their advantages. Industrial development in cities must be clearly defined in the planning stages about who should do what, where, when, and how so that urban spatial plan implementation would be successful and sustainable.

The limited availability of data presents a barrier to conducting further computational analyses. Additionally, the sample size needs to be increased to cover all aspects of urban spatial issues adequately. To gain a comprehensive understanding, further evaluation of spatial land use planning is necessary. Hence, the study recommends further investigation into the evaluation of urban spatial plans both pre- and post-implementation to obtain more quantitative insights regarding their effectiveness. The study also acknowledges the limitation of not being able to conduct Confirmatory Factor Analysis (CFA) to fully comprehend the EFA results. This gap should be addressed in future research endeavors.

## Author contribution statement

Melaku Tanku: Conceived and designed the experiments; Performed the experiments; Analyzed and interpreted the data; Contributed reagents, materials, analysis tools or data; Wrote the paper.

Berhanu Weldetensae: Analyzed and interpreted the data; Wrote the paper.

## Funding statement

This research did not receive any specific grant from funding agencies in the public, commercial, or not-for-profit sectors.

## Data availability statement

Data included in the article are available upon request.

## Additional information

No additional information is available for this paper.

## Declaration of competing interest

The authors declare that they have no known competing financial interests or personal relationships that could have appeared to influence the work reported in this paper.

## Footnotes

1 The FDRE constitution has adopted the federal state structure in 1995 and hence leading to the establishment of a two-tiered government comprising the federal and regional governments. Art 50 (4) of FDRE constitution states that ‘State government shall be established at state and other administrative levels that they find necessary. Adequate power shall be granted to the lowest units of government to enable the people to participate directly in the administration of such units [82].
